# Relative roles of biological and physical processes influencing coral recruitment during the lag phase of reef community recovery

**DOI:** 10.1038/s41598-020-59111-2

**Published:** 2020-02-12

**Authors:** Marine Gouezo, Dawnette Olsudong, Katharina Fabricius, Peter Harrison, Yimnang Golbuu, Christopher Doropoulos

**Affiliations:** 1 Palau International Coral Reef Center, PO Box 7086, Koror, Palau; 2 Australian Institute of Marine Science, PMB 3, Townsville, Q4810 Australia; 3 Marine Ecology Research Centre, Southern Cross University, PO Box 157, Lismore, NSW 2480 Australia; 4 Commonwealth Scientific and Industrial Research Organisation, St Lucia, QLD 4067 Australia

**Keywords:** Community ecology, Marine biology

## Abstract

Following disturbances, corals recolonize space through the process of recruitment consisting of the three phases of propagule supply, settlement, and post-settlement survival. Yet, each phase is influenced by biophysical factors, leading to recruitment success variability through space. To resolve the relative contributions of biophysical factors on coral recruitment, the recovery of a 150 km long coral reefs in Palau was investigated after severe typhoon disturbances. Overall, we found that benthic organisms had a relatively weak interactive influence on larval settlement rates at the scale of individual tiles, with negative effects mainly exerted from high wave exposure for *Acropora* corals. In contrast, juvenile coral densities were well predicted by biophysical drivers, through both direct and indirect pathways. High densities of *Acropora* and Poritidae juveniles were directly explained by the availability of substrata free from space competitors. Juvenile *Montipora* were found in higher densities where coralline algae coverage was high, which occurred at reefs with high wave exposure, while high densities of juvenile Pocilloporidae occurred on structurally complex reefs with high biomass of bioeroder fish. Our findings demonstrate that strengths of biophysical interactions were taxon-specific and had cascading effects on coral recruitment, which need consideration for predicting reef recovery and conservation strategies.

## Introduction

A key objective in spatial ecology is understanding the relative influence and hierarchy of biological and physical processes during recovery^1^. Propagule supply, growth, and survival are key demographic bottlenecks for the recovery of disturbed biogenic habitats and drive the duration of the lag phase of recovery – starting from immediately after disturbance to the beginning of population growth^2,3^. Propagules supply from remnant populations is highly variable in time and space both in terrestrial^4^ and marine systems^5,6^. During early recovery, space limitation is relaxed, and facilitation or inhibition can determine recovery trajectories of habitat-forming organisms^7–9^. However, the interactions effects on propagule settlement and post-settlement success can vary in strength along environmental gradients during early colonization^10–12^. Characterizing the relative influence of biophysical processes driving recovery is therefore a complex but significant undertaking^13,14^, to assess the appropriateness of conservation strategies^15^ as anthropogenic stresses on coastal ecosystems continually increase^16^.

Reef-building scleractinian corals and other benthic organisms that provide structure for entire ecosystems often have a bipartite life cycle, beginning with a dispersive planktonic larval stage, followed by a sessile adult benthic stage^13,17^. Because of global climate change, corals are impacted at increasing frequency and intensity by large scale disturbances, including mass bleaching^18^, predator outbreaks^19^, and storms^20^. Thus, coral reefs often consist of a network of patches at multiple successional states, structured by biological and physical processes, and interconnected by larval dispersal. This study presents a multi-scale investigation of the dominant processes during the lag phase of recovery in a coral reef ecosystem, to better understand the relative influence of temporal, spatial, physical and biological drivers on the re-establishment of corals after disturbances.

Densities of juvenile coral colonies are often used as proxies for recruitment success^21–23^ as their abundance and spatial distribution represent the cumulative outcome from larval supply, settlement and post-settlement processes^24,25^ and often correlate with community recovery trajectories^3,26–28^. Larval supply is variable through time and space, from within reef patches to ecosystem scales. This variability, often referred to as ‘recruitment pulses’^29^ is primarily driven by episodic and patchy larval release combined with variable current forces^30,31^. Once larvae arrive to a reef, coral settlement is affected by a series of ecological interactions occurring at scales from centimeters to kilometers (Fig. S1, ESM Section I). Habitat selection, facilitation, and competition occur during larval settlement. Settlement choice and metamorphosis of larvae are facilitated or inhibited by abiotic (surface roughness and microtopography) and biotic factors (benthic organisms such as crustose coralline algae (CCA) and biofilms) in which the relationships often depends on the interacting taxa^17,32,33^ (ESM Section I.4). Both top-down and bottom-up processes have also been shown to affect coral settlement and post-settlement survival^24,34^, including herbivory that can limit space competition with macroalgae^35^. The physical properties of a reef such as high structural complexity, at micro to meso scales, or low to medium exposure to waves and turbulence and their influences on benthic organisms, are also known to positively affect survival of early-stage corals^12,36,37^. Interactions among these biological and physical processes can have cascading effects on recruitment success, hence identifying their relative importance at different scales is needed for better predicting recovery patterns.

This study occurred on the eastern reefs of Palau with the aim of identifying the relative contributions of biological and physical interaction on coral recruitment at multiple scales during the lag phase of recovery. Eastern Palauan reefs were severely damaged by two super-typhoons in 2012 and 2013, reducing mean live coral cover from ~35% to ~6%^38^. In 2016, live coral cover remained low, ranging between 0.4% and 9% depending on locations and depths (Fig. S2). We focused on four coral groups with distinct functional traits that dominate many Indo-Pacific reefs (*Acropora*, *Montipora*, *Porites* and Pocilloporidae spp.). To characterize whether larval supply or settlement could limit recovery spatially or seasonally, coral larval settlement onto tiles was quantified at nine impacted sites during 6 sampling periods over 2 years. The influence of physical and biological interactions with colonizing organisms on tiles affecting variation in coral settlement were then investigated at the scale of tiles. To examine recruitment success, juvenile coral density (colony diameter ≤ 5 cm) was quantified on the reef benthos at each study site. We then explored juvenile coral density variability and correlations with coral settlement rates. Finally, all findings were incorporated into a structural equation model (SEM) to investigate the hypothetical direct and indirect effects of bio-physical variables on the success of coral recruitment – from settlement to juvenile stages – during the lag phase of reef recovery.

## Results

### Spatio-temporal patterns in coral settlement and tile communities

**Figure 1:**
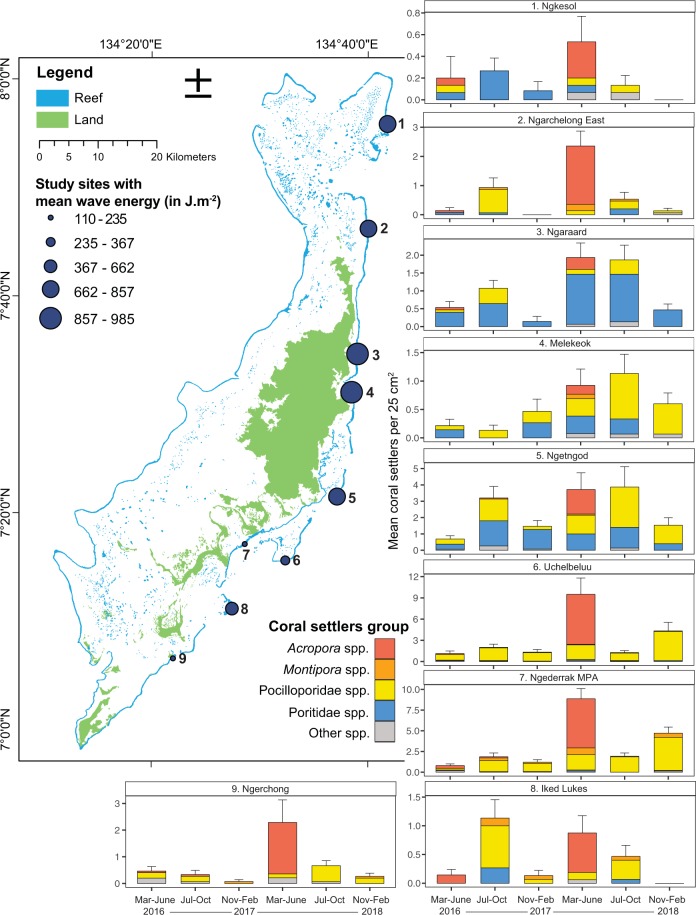
Map of Palau showing study sites, with the mean levels of wave energy (2015–2017), and mean abundance of different group of coral settlers per 25 cm^2^ at each season. Plots are displayed from North to South along the Eastern outer reefs. Note different scales in y-axes among sites.

Across the six sampling periods, a total of 1096 coral settlers were recorded on the underside surfaces of the 791 tiles on the damaged eastern outer reef slope sites. Settlement rates averaged 277 (±81 SEM) settlers.m^−2^.yr^−1^, with up to 5-fold differences in means across sites (Fig. 1). Coral settlement density significantly differed across sites within sampling periods (GLM-NB, significant interaction, P < 0.001, Fig. 1, Table S1). When pooled across sites, settlement was typically highest in the March-June 2017 followed by July–October 2016 and 2017 sampling periods. Settlement was lowest in November-February for both years.

*Acropora* settlement peaked during March-June, and was 20 times higher in 2017 than 2016. Spatially, the central outer reefs, specifically Ngederrak MPA and Uchelbeluu, had the highest *Acropora* settlement rates, with an average of 6.5 (±1.1 SEM) settlers.25 cm^−2^. *Montipora* settlement was generally low with an average of 0.05 (±0.01 SEM) settlers.25 cm^−2^, predominantly found at Ngederrak MPA. Pocilloporidae settlement was more homogeneous throughout the sampling periods but occurred at higher densities in the central outer reefs with 1.5 (±0.1 SEM) settlers.25 cm^−2^. Poritidae settlement predominantly occurred at the central-northern sites displaying an overall mean density of 0.6 (±0.2 SEM) settlers.25 cm^−2^. The reef site Ngetngod had the highest mean densities of Poritidae settlers with 0.95 (±0.3 SEM) settlers.25 cm^−2^, with little variability through time (Fig. 1)

Tile community composition differed across sites x sampling periods (significant interaction, PERMANOVA, P < 0.001, Fig. S3). The first axis of the PCO described 44% of the total community variation and was correlated with crustose coralline algae (CCA) and encrusting fleshy algal (EFA) cover, while the second axis (23% of total variation) was most related to turf algal and colonial invertebrates cover. Cluster analysis revealed two major spatial groupings that were most strongly associated with levels of wave energy. At lower wave energy reefs (Uchelbeluu, Ngederrak MPA and Ngerchong), tile communities were dominated by thick turf, colonial invertebrates such as bryozoans or ascidians, and non-encrusting forms of fleshy macroalgae. In contrast, reefs with medium to high wave energy (Ngkesol, Ngaraard, Ngetngod and Iked Lukes) had tile communities more consistently composed of CCA and EFA, while other organisms were sparse. Tile communities at Ngarchelong East and Melekeok were variable throughout the six sampling periods.

### Predictors of coral settlement

**Figure 2:**
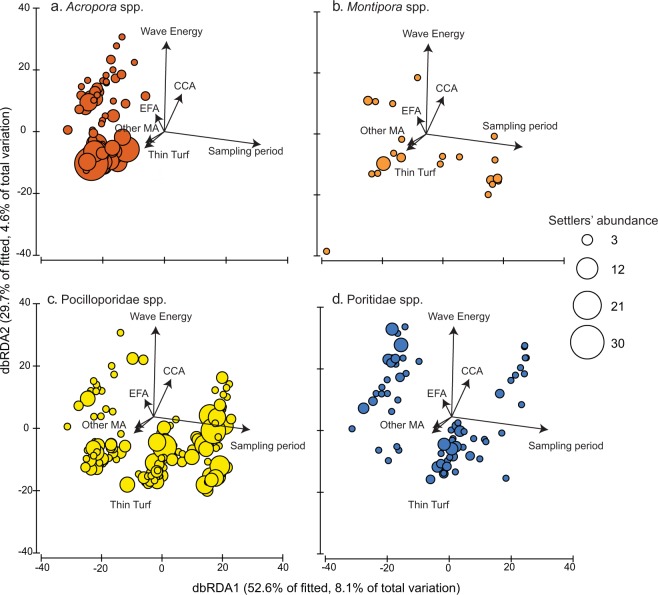
Distance-based redundancy ordination plots between settlers’ density of *Acropora* (**a**), *Montipora* (**b**), Pocilloporidae (**c**) and Poritidae (**d**) during the three sampling periods in 2017 and significant biophysical predictors (tile organisms, wave energy) represented by overlaying vectors. The distance-based redundancy ordination plot was split into 4 plots for visualization.

Physical and biological predictors of coral settlement generally had weak effects (DISTLM, R^2^ = 0.15) and were visually different among coral groups (Fig. 2). Sampling periods had a relative influence of 7% (P < 0.001) on settlement at the community level, followed by wave energy and CCA cover on tiles (4 and 3% relative influence, respectively; P < 0.001 for both). Ordination plots show the seasonal peak of *Acropora* settlement (along the x-axis), as well as the effect of wave exposure along the y-axis (Figs. 2 & S4). CCA on tiles only had a positive effect on *Acropora* settlement up to 12% cover (Fig. S4). For other coral groups, settlers’ abundances were scattered on ordination plots, indicating no effects from the studied variables (Fig. 2).

### Spatio-temporal patterns in juvenile coral densities

**Figure 3:**
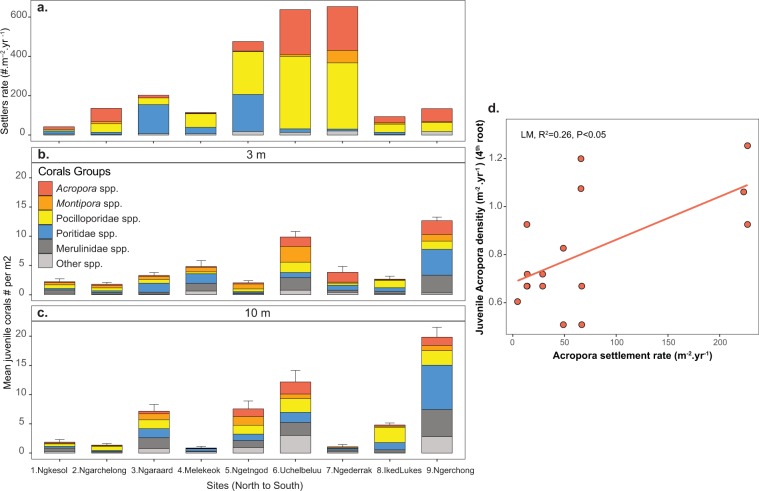
Settler rates on tiles (m^−2^.year^−1^) of major coral groups (**a**), and densities of juvenile corals (averaged of year 2016 and 2017) at 3 and 10 m depth (n = 5 transects). (**b**,**c**) Error bars show one standard error of total juvenile densities. Regression plot showing relationship between the cumulative settlement rate for *Acropora* corals from 2016–2017 with the densities of juvenile *Acropora* in 2017 (size 0.5–5 cm).

Across all sites and both years, total juvenile coral densities averaged 4.8 (±0.4 SEM) colonies.m^−2^ at 3 m, and 6.3 (±0.7 SEM) at 10 m depth (Fig. 3). Total density showed a significant 3-way interaction between years, sites and depths (GLM-NB, P < 0.01, Tables S2–3 Fig. S5). During both years and at both depths, juvenile coral density was highest at Ngerchong, which contrasted with coral larval settlement patterns, followed by Uchelbeluu, similar to coral settlement patterns on tiles. Juvenile coral density was lowest at the two northern sites.

### Predictors of juvenile coral abundance

When investigating relationships between different size classes of juvenile corals in 2017 and settlement rates from 2016 and from both years (2016–2017), we found significant but variable relationships (R^2^ = 0.26) between juvenile *Acropora* density (0.5–4 cm and 0.5–5 cm sizes) and *Acropora* settlement density from 2016–2017 (Fig. 3, Table S4). No significant relationships between settlement rates and juvenile densities were found for other coral groups.

**Figure 4:**
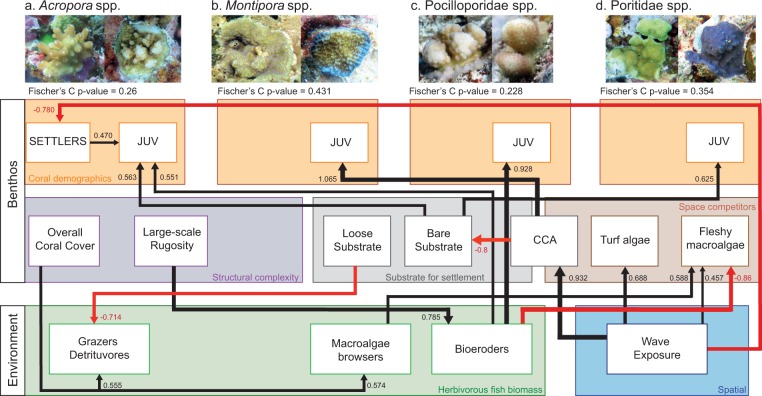
Four structural equation models (SEM) showing the direct and indirect effects of coral demographics, ecological and spatial variables on the abundance of *Acropora* (**a**), *Montipora* (**b**), Pocilloporidae (**c,d**) Poritidae juvenile corals during the early phase of recovery. SEM analyses were run separately for each coral group but are presented on the same diagram. The thickness of paths is proportional to the given standardized path coefficients but cannot be compared among coral groups. Black and red arrows indicate positive and negative pathways, respectively. Non-significant pathways and variables are not shown.

Findings from the structural equation modelling (SEM) support the hypothetic pathways inferred by the initial path diagram (Fig. S1) for juvenile coral densities from all groups (Fisher’s C statistic p-values >0.22, Fig. 4). Conditional R^2^ are described for each additive component model when significant, whereas path coefficient values of predictors for each SEM are shown within Fig. 4. For *Acropora* and Poritidae, juvenile densities were positively correlated with the cover of bare substrate (Conditional R^2^ = 0.77 and 0.43 respectively), which was negatively correlated to CCA cover (R^2^ = 0.57) occurring at low exposure reefs. In parallel, *Acropora* juvenile corals were more abundant at sites with high *Acropora* settlers (R^2^ = 0.77), and both stages were negatively related to wave energy (R^2^ = 0.68). *Montipora* juvenile corals were positively affected by CCA cover (R^2^ = 0.49), which was positively related to wave exposure (R^2^ = 0.77). Direct positive relationships were evident between the biomass of bioeroding fish and juvenile densities of *Acropora* (R^2^ = 0.77) and Pocilloporidae (R^2^ = 0.38).

SEMs revealed additional interaction occurring throughout the study system. Reefs with high wave exposure were characterized by high CCA, turf and macroalgae coverage. Although fleshy macroalgae cover remained low (<4%), it was negatively related to bioeroding fish but attracted macroalgae browsers. All herbivorous fish biomass except scrapers were positively influenced by the increasing structural complexity, both through increased live coral cover or abiotic structure. There were no relationships detected between live coral cover and coral settlers per 25 cm^2^ or juvenile densities on reefs, suggesting low rates of larval retention and ‘self-seeding’.

**Figure 5:**
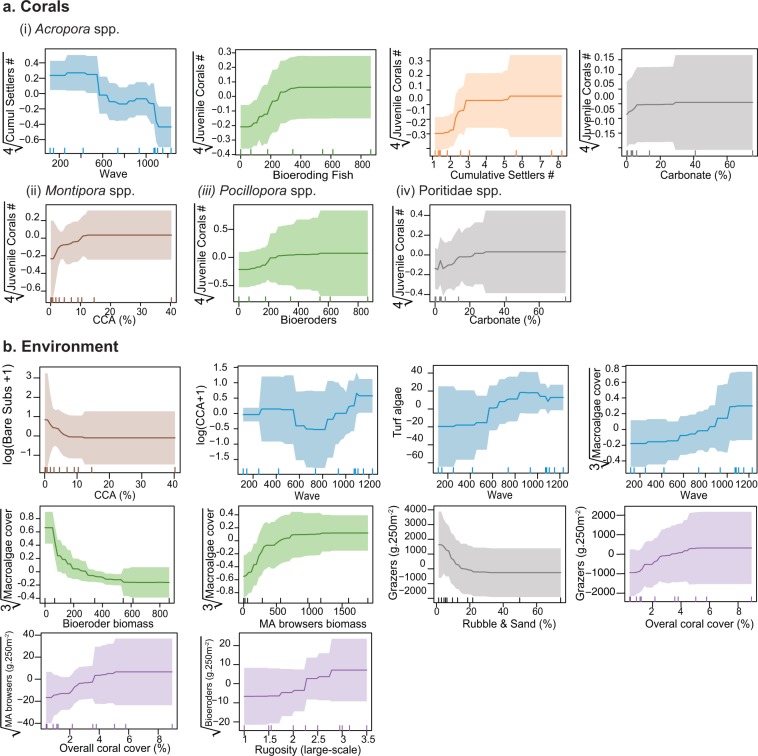
Shape of relationships detected by the SEM using ABTs for corals (**a**) and environmental response variables. (**b**) Colors match the color of group of explanatory variables (Fig. S1), blue = waves, green = herbivory, orange = coral demographics, grey = substrate for settlement, brown = space competitors, purple = structural complexity.

Subsequent aggregated boosted trees (ABT) analyses on variable relationships detected by the SEM highlighted threshold values as well as non-linear relationships (Fig. 5a,b). There was a positive density-dependent effect of *Acropora* settlement on *Acropora* juvenile density from 0–3 settlers.25 cm^−2^, after which there was no effect. The effect of CCA on *Montipora* juvenile corals was positive, but only up to ~12% CCA cover and CCA cover only increased with wave energy at levels >800 J.m^−2^. The effect of bioeroding fish on juvenile corals was positive up to a biomass of ~400 g.250 m^−2^. The effect of macroalgae browsers on macroalgal coverage slowed down when biomass was >~750 g.250 m^−2^. Lastly, the cover of loose substrate negatively influenced the biomass of grazers especially when ≥20%.

## Discussion

The influence of biophysical interactions on recruitment can be variable through time and space, often leading to unpredictable effects on recruitment patterns. Our findings show that, overall, processes driving recruitment success were hierarchal, often indirect, and occurred at different spatial scales. Coral settlement was found to be highly variable through time and space, as shown in previous studies^39–41^. Such variability highlights the common occurrence of recruitment pulses driven by larval supply in marine ecosystems, as settlement rates were only weakly explained by biophysical predictors at the scale of the tiles. We also found that other processes facilitated recovery following larval supply, such as the direct role of bare substrate availability and indirect role of wave energy influencing benthic communities and subsequent larval settlement, and reef structural complexity influencing the biomass of herbivorous fish. Below, we discuss in detail the processes influencing coral larval settlement and recruitment patterns at different scales, from the scale of settlement tiles (0.0025 m^2^) to the area of the belt transects (250 m^2^) to the reef system spanning 150 km.

In this study, coral larval settlement assessments integrated larval supply and settlement. The correlation between coral settlement and commonly described facilitative and inhibitive organisms on tiles was found to be relatively weak. Thus, the contrast in settlement rates between coral groups likely resulted from their taxon-specific reproductive traits, larval dispersal potential, and hydrodynamic forcing^30^.

Seasonal differences in reproduction among the coral groups were reflected in the larval settlement data for *Acropora* spp., which predominantly settled in the March-June periods, but not for the three other coral groups. Previous data from Palau demonstrated multiple spawning events occurring throughout the year depending on taxa, and *Acropora* spawning events consistently occurring from March to May^42^. Annual differences in settlement were also pronounced for *Acropora*, but not for the other settler groups. *Acropora* settlement was 20 times higher in 2017 than 2016, likely caused by variable current induced larval dispersal^43,44^. However, identifying hydrodynamic conditions that relate to a recruitment pulse remains challenging as it relies on long-term settlement data. For instance, 23 years of recruitment data was needed to detect that specific wind conditions following the spawning of a snapper species was associated with high recruitment rates on a reef^45^.

Interestingly, we found a positive but variable association (R^2^ = 0.26) between juvenile densities in 2017 and larval settlement rates from both years for *Acropora*, but not for the other coral groups, possibly partially reflecting the relatively fast growth rates of *Acropora* spats and juveniles^25^. Predicted larval supply was also found to be positively correlated with densities of juvenile coral and recovery rate following mass bleaching disturbance for *Acropora* corals^3,46^. Together, these results demonstrate that recruitment success of the fast growing *Acropora* spp. may largely be driven by larval supply, as also demonstrated in larval restoration work^25^. Therefore, larval supply appears to be a good predictor for the recovery of this ecologically important coral genus, unlike the other studied taxa.

Pocilloporid corals often display an opportunistic life strategy^47^, in part because of their dominant reproductive mode as brooders, generally fast growth rates, and small size to sexual maturity (5–8 cm). Given the predominant brooder reproductive mode of Pocilloporidae in Palau, with colonies gaining maturity at small size classes and releasing planulae that typically have localised dispersal, a relationship between settlers on tiles and juveniles on the benthos was expected^21,23,48^. However, in contrast to *Acropora* corals, no relationship between Pocilloporidae settlers and juveniles was observed in our study (Table S4), even though settlement rates were similar or higher than the densities observed for *Acropora*. We postulate that settlement failure for Pocilloporidae larvae on natural substrata and/or high post-settlement mortality may have resulted in this decoupling. Processes causing post-settlement mortality may be driven by competitive and inhibitive species interactions such as with turf algae^12,24^ (but this effect would have to be stronger on Pocilloporidae than *Acropora*), high rates of predation on Pocilloporid recruits^49^, and/or larvae settling unselectively on rubble fields that occurred in high coverage (up to 75%) depending on studied sites and depths. For the latest, *Acropora* larvae may likely be more selective of their microhabitat during settlement e.g.^50^ than Pocilloporidae larvae, and therefore, the mechanic instability of rubble substrata would cause mortality of young Pocilloporid corals^51^. Our finding implies that in our study system where *Acropora* and Pocilloporidae settlement rates were found to be similar, but juvenile densities were not, *Acropora* may have a competitive advantage over Pocilloporidae corals during early post-settlement stages.

Our findings also show that the northern outer reef locations consistently had low settler and juvenile coral densities. This finding implies that this portion of the outer reefs might be limited by larval supply due to hydrodynamic conditions that prevailed during this study. The northern outer reefs are highly exposed to easterly wind and swells from December to May, which may have decreased larval retention. In addition, the infrequent reef connectivity with the island of Yap^46^, was probably not captured during our study timeframe.

While our coral larval settlement assessment provided useful insights in regards to larval supply patterns through space and time, especially for the fast growing *Acropora* corals, the method has some limitations for other taxa. First, there can be up to a 3–4 years lag between a recruitment pulse and the time when juvenile corals become visible on the reef ^52^ but this is highly dependent on coral taxa and their growth rates. This specifically applies to the poritid taxa, which exhibit high offspring survival but slow growth^40,53^. Interestingly, our study site ‘Ngerchong’ had the highest juvenile coral densities (including poritids) but had a low larval settlement rate on tiles during the study period, including minimal poritid settlers. Furthermore, settlement tiles may not be conducive to settlement of some coral taxa such as the family Merulinidae as they are rarely observed to settle on tiles^54,55^ despite their occurrence on reefs at both juvenile and adult stages. Finally, we highlight the need to explain the inconsistency in larval supply patterns through time at the coral community level. Advances in the development of hydrodynamic models in combination with *in situ* coral settlement assessments following spawning events are needed to better understand drivers of recruitment pulses on reefs. Improvement of coral settlement assessment methodologies could also be made using underwater microscopic imaging^56^, fluorescence techniques^57^ and/or the development of alternative tiles that enable settlement of all taxa.

Wave energy was found to strongly influence the community of benthic organisms both on the settlement tiles and on the reef and had strong relationships with coral recruitment patterns, as also shown by Edmunds et al.^58^, although the direction of these relationships differed among coral taxa in our study. Two of the three sites with tile communities typical of low wave energy environments had the highest *Acropora* settlement. As settlement tiles were conditioned at the sites prior to spawning, this indicates that *Acropora* corals may be sensitive to the dynamic environment of highly exposed reefs at early life stages. Increasing wave energy can also increase the productivity and competitive abilities of CCA and turf algae^59^, as well as reducing interaction strength between herbivorous fish on primary producers, resulting in cascading effects on coral settlement^12^. In this study, juvenile densities of *Acropora* and *Porites* were highest where high coverage of bare substrata (≥30%) was present, free from high abundance of competitors. In contrast, the density of *Montipora* juveniles on the benthos was positively influenced by the cover of CCA. High cover of CCA occurred at highly exposed reef sites with wave energy >800 J.m^−2^. It is likely that in high wave energy environment, *Montipora* colonies, occurring mostly as encrusting morphotypes, are better adapted to withstand the mechanical stress from high wave action^60^ as opposed to branching or tabular *Acropora* corals for example. Additionally, the survivorship of *Montipora* corals may also be favored where the abundance of fouling algae are low^61^, typical of reef habitat with high wave action and herbivory levels, as both mechanical and grazing forces prevent fouling by other algae^62^

Our study results highlight some interesting properties of our studied system that were associated with increased recruitment and hence expecting optimal coral recovery. Three of the four studied taxonomic groups of juvenile corals were found in higher density where the substrate was either relatively free from competitors or had high CCA cover. In addition to wave energy, herbivory was a strong predictor for some of the recruitment patterns. Bioeroding fish were found with higher biomass at sites with high structural complexity, and they were positively associated (even at a low biomass threshold of 200 g.250 m^−2^) with the density of *Acropora* and Pocilloporidae juveniles and negatively associated with the cover of fleshy macroalgae. This finding suggests that the release of competition from fleshy macroalgae outweighs the potential predatory effect on corals by this fish group^63–65^. The low cover of fleshy macroalgae (<4%) indicates top-down control of macroalgae by herbivory, which should be maintained to maximize recovery potential. Although wave energy can influence herbivorous fish species composition^66^, this was not detected at the larger spatial scale of our study. Instead, the structural complexity of reefs, provided by live coral remnants or abiotic structure, was the main factor influencing the biomass of different functional groups of herbivorous fish^67^.

A previous study in Palau have shown that coral reef recovery occurred within 9–12 years after 1998 mass bleaching event, but that was evident only for the western outer reefs and inner bay reefs^3^. The eastern outer reefs were not fully reassembled from the mass bleaching event when severe damage was incurred by the typhoons within this habitat^3,38^. Five years have passed since the typhoon disturbances and coral coverage remains below 10% (Figure S2), highlighting that recovery following intense storms likely take longer than following bleaching event^6,68^ due to the additional structural damage on substrata caused by waves. As shown by our analyses, the length of the lag phase of recovery following a disturbance is context-specific of the reef location in space, and requires consideration when managing reefs during recovery. Therefore, we listed threshold values of the tested biophysical drivers, that were found to significantly influence the success of recruitment (Fig. 5) in our study system, depending on coral taxa (Table 1). Such values can be used for comparison by other studies or as targets when managing reefs for recovery to optimize coral recruitment success

**Table 1:** Showing threshold values extracted from Fig. 5 that had a significant and positive effect on coral recruitment success depending on coral taxa.

Recovery drivers	Threshold values for a positive effect on recruitment success	Coral taxa
Settlers	70–100 settlers.m^−2^.year^−1^	*Acropora*
Wave energy	<600 J.m^−2^	*Acropora*
Bioeroding fish biomass	0.8–1.2 g.m^−2^	Acropora, Pocilloporidae
CCA cover	3–12%	*Montipora*
Hard substrata free from space competitors	≥30%	Poritidae

To conclude, our study shows that hierarchal processes occurring at several scales drove the success of coral recruitment during the lag phase of recovery after corals were decimated by consecutive super-typhoon disturbances. We found that benthic organisms exerted a relatively weak interactive influence on larval settlement rates at the scale of individual tiles, with negative effects mainly exerted from high wave exposure for *Acropora* corals. This finding is driven from biophysical interactions occurring on artificial tile substrata, which may differ on natural substrata. The positive association found between juveniles and settlers for *Acropora*, highlights the role of larval supply in the recovery of this key reef-building coral. Therefore, we stress the need to develop a better proxy to quantify and understand larval supply at the community level, as well as identifying locations that are consistently larval supply-limited through time. Following larval supply, the availability of bare substrate, the levels of wave energy and the structural complexity of reefs affected juvenile coral densities, through direct and indirect pathways. Therefore, we encourage future studies to address the hierarchy of processes influencing coral recruitment occurring at different spatial scales. Such studies are important to better identify the roles of these factors in driving coral recruitment success across different systems, and will improve our capacity to predict recovery at ecosystem scales.

## Methods

Two types of field surveys were conducted at nine sites (Fig. 1) as detailed below.

### Spatio-temporal patterns of coral settlement and colonizing organisms on tiles

To assess coral larval supply and settlement rates at the study sites over two years, we used methods of tile assemblage and deployment as described in Doropoulos *et al*.^69^ (Fig. S6). At each site, 15 unconditioned tiles were positioned ~1 meter apart at 7–8 m depth and ~5 cm above the substrate to create a habitat favored for coral settlement^70^. They were attached with nylon cable ties to concrete nails hammered into hard dead reef substrates. Tiles were exchanged every 4 months from February 2016 to February 2018 (6 sampling periods) to capture major coral spawning events, known to occur mainly from March to June and from August to October^42^. Upon retrieval, tiles were retained in seawater, gently rinsed to remove fine sediments, and photographed at high definition for tile community cover analysis. Tiles were then placed into a 10% bleach solution for 24 h, rinsed under running freshwater and dried. Bleached and dried tiles were inspected for coral settler densities, classified into the following groups: *Acropora* spp., *Montipora* spp., Pocilloporidae spp., Poritidae spp. and ‘Other species’ using Baird and Babcock^71^ Babcock *et al*.^72^. As ~90% of the settlement occurred on the underside surfaces of tiles in 2016, only settlement onto undersides was quantified in 2017. Settlement rates on the underside surfaces of the tiles are presented in this study.

To quantify the cover of organisms settled on the underside surface of tiles, photos were analyzed with CPCe software^73^ using 50 random points per photo classified into the following 11 categories were used: biofilm tile, CCA, thick turf algae, thin turf algae, encrusting fleshy macroalgae (EFA), other macroalgae, bryozoans, ascidians, sponges, spiral worms, and ‘other invertebrates’. Thick turfs consisted of thick green and brown algal mats or small red algae that were potentially associated with either trapping sediments or overgrowing coral recruits. Thin turfs consisted of very sparse green or brown algal patches amongst which coral larvae could settle.

### Coral reef community surveys and wave exposure

Ecological surveys of reef communities were conducted at the same nine study sites in 2016 following previously described methods^3,38,74^ along five 50-m long GPS-located but unmarked transects at 3 and 10 m depths. Benthic cover, juvenile coral density (≤5 cm) and fish sizes and abundances were recorded. Juvenile coral surveys were repeated in 2017. Reef structural complexity was quantified at two scales at each site and depth. Large-scale rugosity was based on a visual assessment of the reef topography (0 to 5 grade)^75^, while small-scale rugosity estimates were based on measurements of a 2-m long chain with ~1 cm links laid parallel to each transect at 10 meter intervals.

The wave exposure of each site during each sampling interval was calculated from wind speed, fetch distance to the reef and angle of exposure to the wind using GIS methods detailed in Houk *et al*.^76^. Satellite derived daily surface wind data from Advanced Scatterometer (ASCAT METOP-A) were downloaded from the Asia-Pacific Data Research Center, from January 2015 (a year before the first juvenile corals survey) to February 2018 (the end of the coral recruitment survey) (url: http://apdrc.soest.hawaii.edu/las/v6/dataset?catitem = 12490).

### Data analyses

Spatial and temporal differences in total coral settlers per 25 cm^2^ (count data) were analyzed using a generalized linear model (GLM, negative binomial (NB) distribution), including sites, sampling periods, and coral groups, and their interactions, as predictors. Assessment of over-dispersion and model validation was conducted following Zuur and Ieno^77^. Results indicated a significant 3-way interaction, therefore, subsequent analyses were performed for each coral group separately.

To investigate spatial and temporal differences in settlement tile community composition, permutational multivariate analysis of variance (PERMANOVA)^78^ was conducted. We examined the effect of sites and sampling periods and their interactions on log +1 transformed Bray-Curtis dissimilarity matrix of tile communities after 999 permutations, followed by pairwise comparisons. Differences in tile community structure were visualized using a principal coordinate analysis (PCO) with vector plots based on Pearson correlations >0.3 to illustrate the relationships between the tile community and the PCO axes. A hierarchical cluster analysis (SIMPROF Test) was performed to show groupings of samples with >80% resemblance. To investigate the interactive effects from tile organisms, wave exposure and sampling period on the community of coral settlers (4 taxa), we used distance-based linear models (DISTLM) on a fourth root transformed Bray-Curtis dissimilarity matrix. As the analysis was conducted at the tile level and tiles in 2016 were not labeled, only data from the year 2017 were used. Model selection was done using the ‘Best’ routine by comparing models’ AIC and BIC. The ‘Best’ model results were visualized using distance-based redundancy (dbRDA) ordination plot. As the data did not meet the assumptions for homogeneity of dispersion (tested using PERMDISP) for both PERMANOVA and DISTLM analyses, a conservative α value of 0.01 was used to avoid a Type I error^79^. Aggregated Boosted Trees (ABT)^80^ were used to further investigate the relationships strengths and shape between the effects detected by the DISTLM model and settlers density for each coral group separately.

Spatio-temporal differences in juvenile coral densities were also investigated between the two studied years, sites and depths with a GLM-NB, followed by pairwise comparison between the two studied years at each site and depth using the function ‘lsmeans’. We then explored relationships between 2017 juvenile coral densities and both 2016 and 2016–2017 coral settlement rate, since juvenile corals surveys were done 4–6 months after major spawning events in 2017. Analyses were repeated for different size classes of juvenile corals considering their growth rate at early stages:^25,48,81^ 0.5–5 cm for *Acropora* spp., 0.5–3 cm pocilloporids and 0.5–2 cm for poritids. Two outliers were removed because of obvious recruitment failure due to high percentage of rubble and sand substrata.

To explore the relative roles and hierarchy of all biophysical drivers on the success of recruitment during the lag phase of reef community recovery, findings were combined from the analyses described above and developed into a structural equation model (SEM). Each path presented in Figure S1 is based on known relationships from the literature, as detailed in the Introduction and ESM. One SEM analysis per coral group was performed. We used the mean juvenile coral densities between the two years, as they were not consistently similar between two years at each site and depth (Fig. S4, Table S3). The variable ‘settlers’ represents the cumulative number of coral settlers among the 6 sampling periods for each site. We assumed that larval supply and settlement was similar at 3 and 10 m because of evidence suggesting that early stage coral survivorship is non-dependent on depth but on habitat properties^82,83^, all accounted for in the SEM model (Figure S1).  Loose substrate was composed of rubble and sand. Bare substrate includes very fine turf cover. Daily wind conditions from January 2015 to January 2017 were used to calculate wave exposure levels to capture conditions from a year to two years before juvenile coral surveys. The predatory effect from herbivores on coral settlers was not included as on the cryptic underside surface of the tile, competition outweighs predation^24^. All SEM component models were linear mixed-effects models (LMM) including depth as a random effect, apart from settlers that were only quantified at one depth, hence this component was based on the linear model. Response variables were transformed when necessary to improve the normality of model residuals. Explanatory variables were scaled during the analytic procedure in the function sem.coefs^84^. Benthic variables are naturally collinear, and were only added to models when suggested as missing links and if the variation inflation factor remained low (<5). The goodness of fit of SEMs were assessed using a directional separation test (d-separations test) Fisher’s C statistic^84^. Once relationships were detected within the SEM framework, details on the shapes of the relationships among variables were explored using ABT^80^.

Univariate and SEM analyses were conducted using R version 3.5.2^85^ using lme4^86^, lsmeans^87^, abt^80^, and piecewiseSEM^84^ packages. Multivariate analyses were conducted using PRIMER-E v.6 with PERMANOVA extension^88,89^

## Supplementary information

Electronic Supplemental Material.
