# Supplementary material for: Relative roles of biological and physical processes influencing coral recruitment during the lag phase of reef community recovery
Source: Sci Rep. 2020 Feb 12;10:2471. doi: 10.1038/s41598-020-59111-2 (PMC7015914; doi:10.1038/s41598-020-59111-2)
Supplement: Supplementary file 1 — Electronic Supplemental Material. [file 41598_2020_59111_MOESM1_ESM.docx]

**Electronic Supplemental Material**

Relative roles of biological and physical processes influencing coral recruitment during the lag phase of reef community recovery

Marine Gouezo^1,3^, Dawnette Olsudong^1^, Katharina Fabricius^2^, Peter Harrison^3^, Yimnang Golbuu^1^, Christopher Doropoulos^4^

^1^: Palau International Coral Reef Center, PO Box 7086, Koror, Palau. [mgouezo@picrc.org](mailto:mgouezo@picrc.org), [dolsudong@picrc.org](mailto:dolsudong@picrc.org) , [ygolbuu@picrc.org](mailto:ygolbuu@picrc.org)

^2^: Australian Institute of Marine Science, PMB 3, Townsville Q4810, Australia. [k.fabricius@aims.gov.au](mailto:k.fabricius@aims.gov.au)

^3^: Marine Ecology Research Centre, Southern Cross University, PO Box 157, Lismore NSW 2480, Australia. [peter.harrison@scu.edu.au](mailto:peter.harrison@scu.edu.au)

^4^: Commonwealth Scientific and Industrial Research Organisation, St Lucia, QLD, 4067, Australia. [christopher.doropoulos@csiro.au](mailto:christopher.doropoulos@csiro.au)

1. **Detailed explanation on SEM paths**


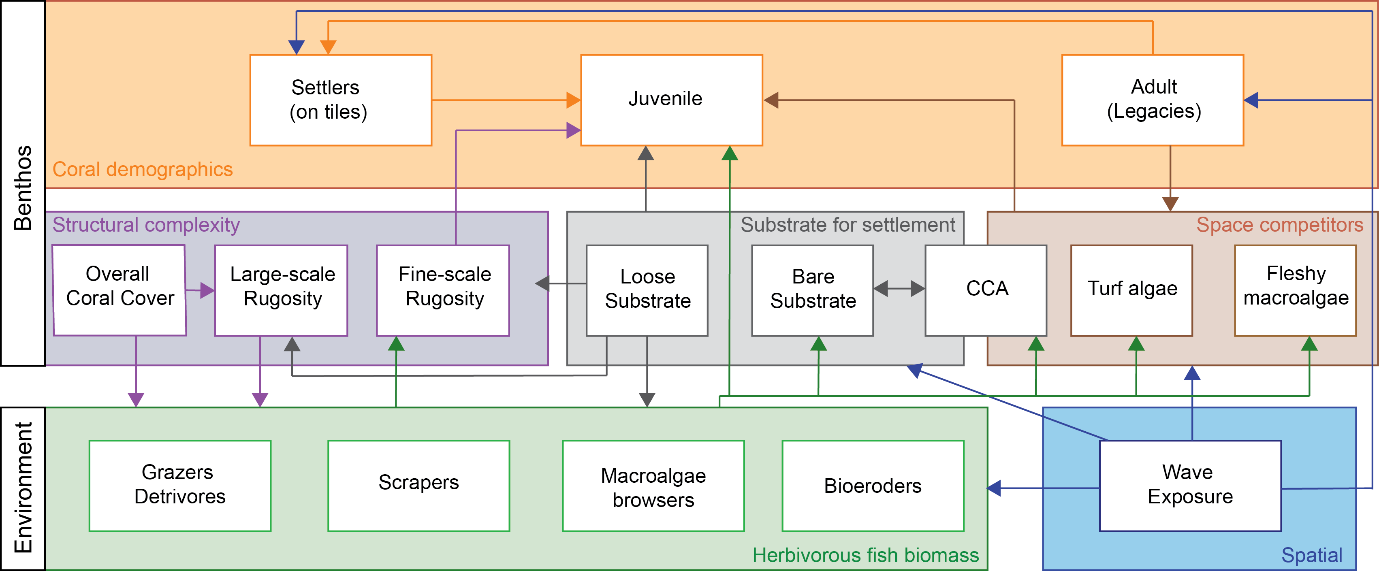


**Figure S1**: Structural equation model (SEM) diagram showing relationships among variables of processes occurring during the lag phase of recovery at a specific point in time. Each path represents a model component. Arrows pointing at or originating from the large colored boxes show that relationships are occurring for all the variables within the box.

1. **Coral demographics processes**: *Settlers* density is affected by the coverage of *adult legacies*; *Juvenile corals* density is affected by the density of *settlers*

In marine systems and especially coral reefs, the migration of foundation organisms that arrive to the disturbed community is highly influenced by the dispersive potential of larvae. This trait allow individuals to recruit into new spaces^1^. The length of larval pelagic phase depends on the reproductive mode of corals (spawners vs brooders)^2^ and on the time needed to acquire competency to settle^3^. The adult legacies remaining after a disturbance may influence the densities of settlers at the reef location if larvae are self-seeded or within the system, if the system is not well connected to undisturbed reefs.

1. **Structural complexity**: *Herbivorous fish biomass* is affected by the *structural complexity of reefs* (made by live coral coverage and reef structure); the *juvenile corals* density is affected by the *fine scale rugosity* of reefs.

The level of structural complexity on coral reefs, often referred to as reef rugosity, has been shown to be positively correlated with reef fish abundance, biomass and diversity ^4–6^. Small crevices on the benthos have been shown to provide refugia from predation for coral recruits ^7^.

1. **Substrate for settlement**: *Juvenile corals* density is directly affected by the *substrata* (made of lose or bare substrata or covered with CCA)

Following dispersal and when larvae become competent to settle, coral larvae must find a suitable substrate to attach and metamorphose then settle permanently. Although many factors influence the choice of substrate attachment by coral planula such as light, depth, water motion, substrate orientation or sedimentation^8,9^, attachment is primarily triggered by chemical and surface properties of the substrata: algal biofilms, bacteria and CCAs^9,10^.

1. **Space competitors**: *Juvenile corals* density is directly affected by the coverage of *space competitors* (CCA, turf alga and fleshy macroalgae)

Upon settlement, young corals compete with other benthic organisms. CCA living on hard substrates may increase coral survivorship through several processes by: (1) inducing settlement and metamorphosis^11^, (2) excluding other space competitors, and (3) providing shelter against sediment as opposed to turf algae that traps sediment^12^. However, not all species of CCA have these abilities. In fact, some CCA species were shown to increase mortality rates of coral settlers by overgrowing them or shedding layers thereby reduction recruitment of corals^13^. Macroalgae have been shown to have negative effects on coral settlement but the extent of this effect varies among taxa^14^. Algal turfs were shown to have the least negative effects compared to upright macroalgae^15^. However, Arnold et al.^16^ demonstrated that turf algae can have inhibitory effects of coral settlement in their herbivory exclusion study^7,16^.

1. **Spatial**: Space competitors, substrata and herbivorous fish community is affected by the level of wave energy

The degree of wave energy on reefs influences primary productivity^17^ and was also found to be associated with macroalgal phase-shift^18,19^, preventing successful coral settlement and recruitment^20^. Wave exposure was also shown to influence the functional make-up of herbivorous fish community^21^.

1. **Herbivorous fish biomass:** *Juvenile corals* density is directly affected by *herbivorous fish*; *substrata* and *space competitors* are directly affected by *herbivorous fish*

Algae assemblages on damaged reefs can vary enormously and consequently have very different effects in successional processes at different spatial scales^14^. Succession among benthic algae usually start from microbial film and cyanobacteria, to thin filamentous algae, to more complex turf and macrophytes^22^. Whether successional algae are turf algae or upright fleshy macroalgae mostly depends on herbivory levels^23–25^. While herbivory limits the competitive ability of macroalgae^26^, some herbivorous fish groups such as scrapers or bioeroders can have predatory effects on coral recruits^27^. However, the net effect of herbivory enhances recruitment rather than reducing it^25,28,29^.

1. **Additional Supplementary Figures**


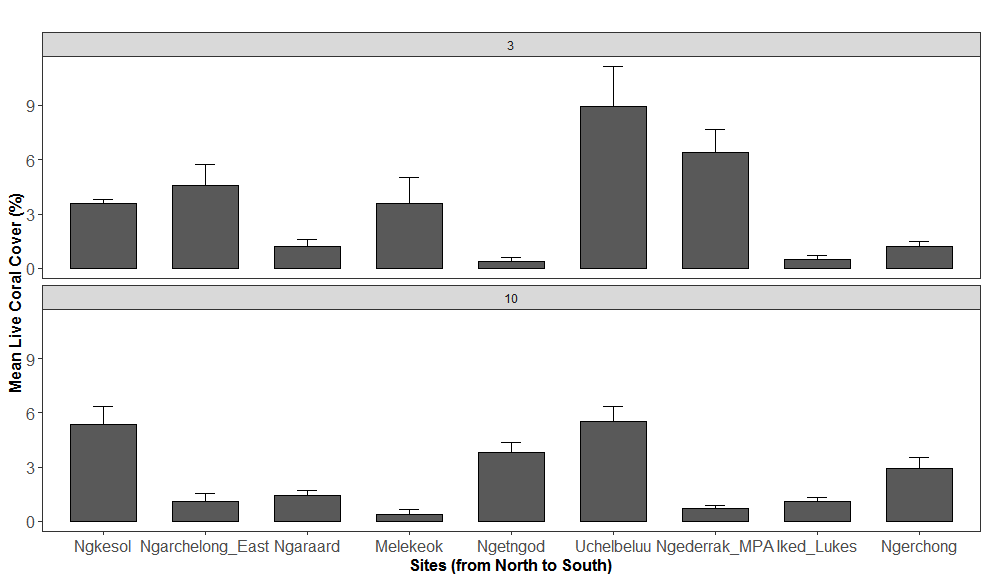


**Figure S2:** Live coral cover percentage recorded in 2016 at 3 and 10 m depth (n = 5 transects). Error bars show one standard error.


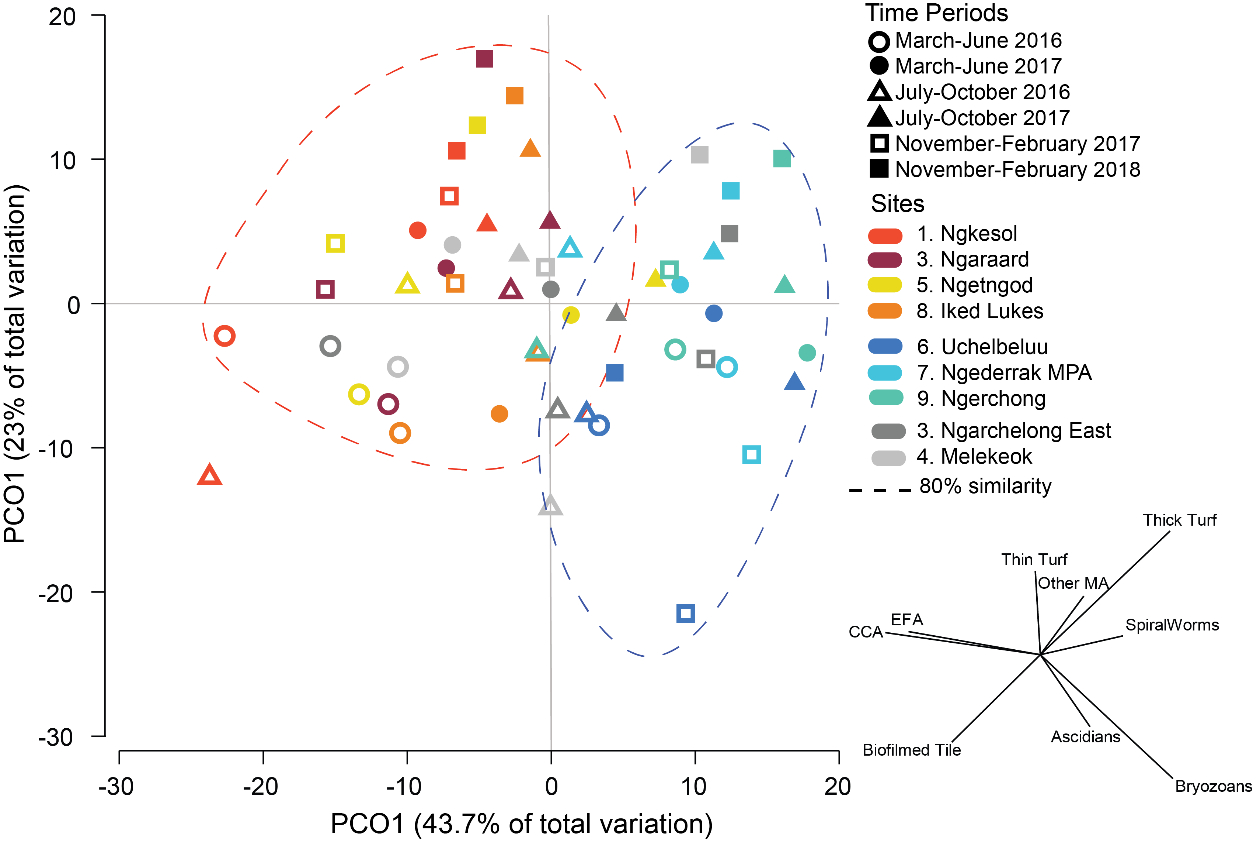


**Figure S3**: Principal Coordinate Analysis (PCO) of tile communities across seasons and sites. Vector overlays represent correlations > 0.3 based on Pearson ranking. Dashed line show site groupings with more than 80 % similarity


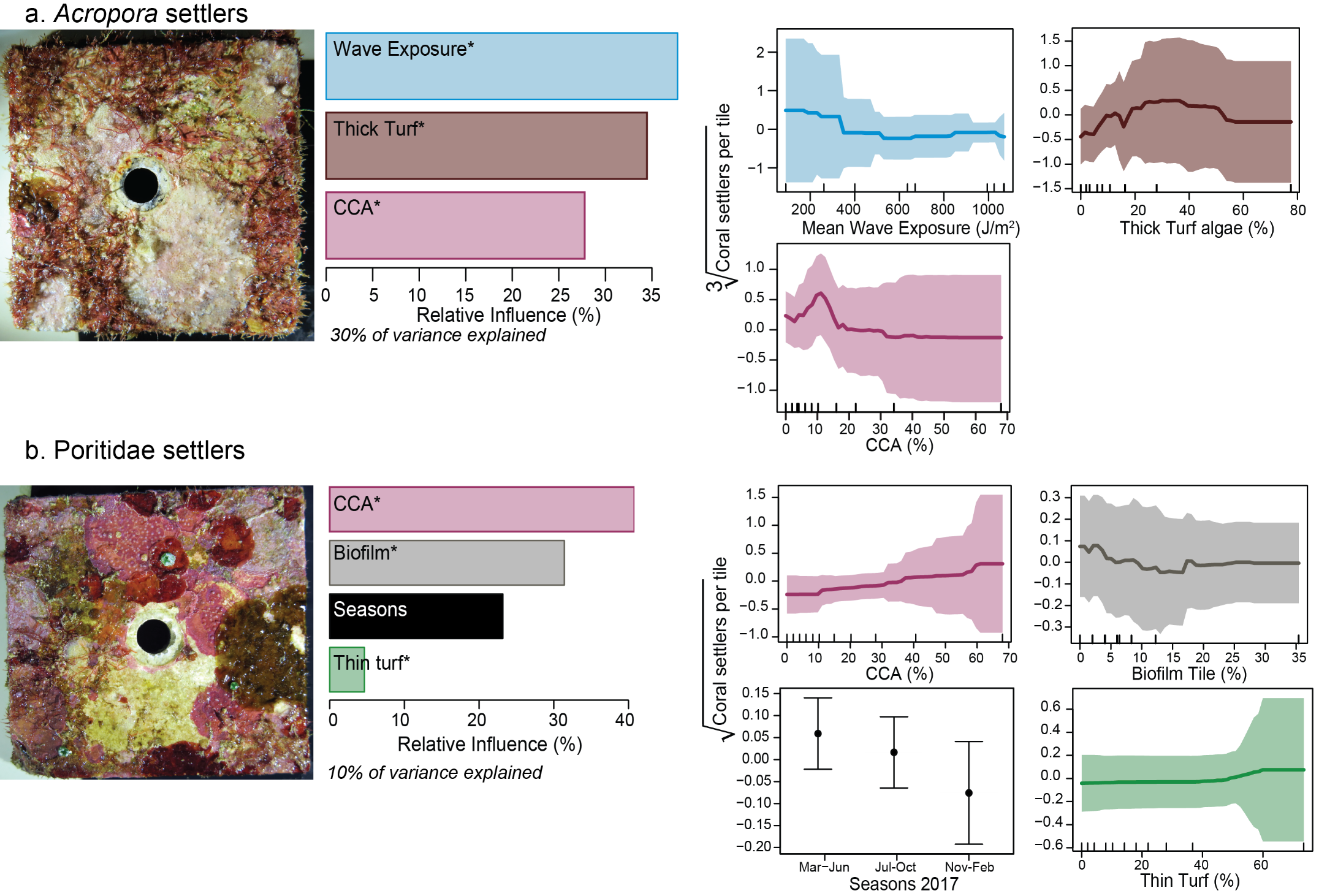


**Figure S4**: Aggregated boosted tree partial effect plots showing the relationships between *Acropora* settler densities and the significant predictors detected by DISTLM: wave exposure and CCA cover on the tile


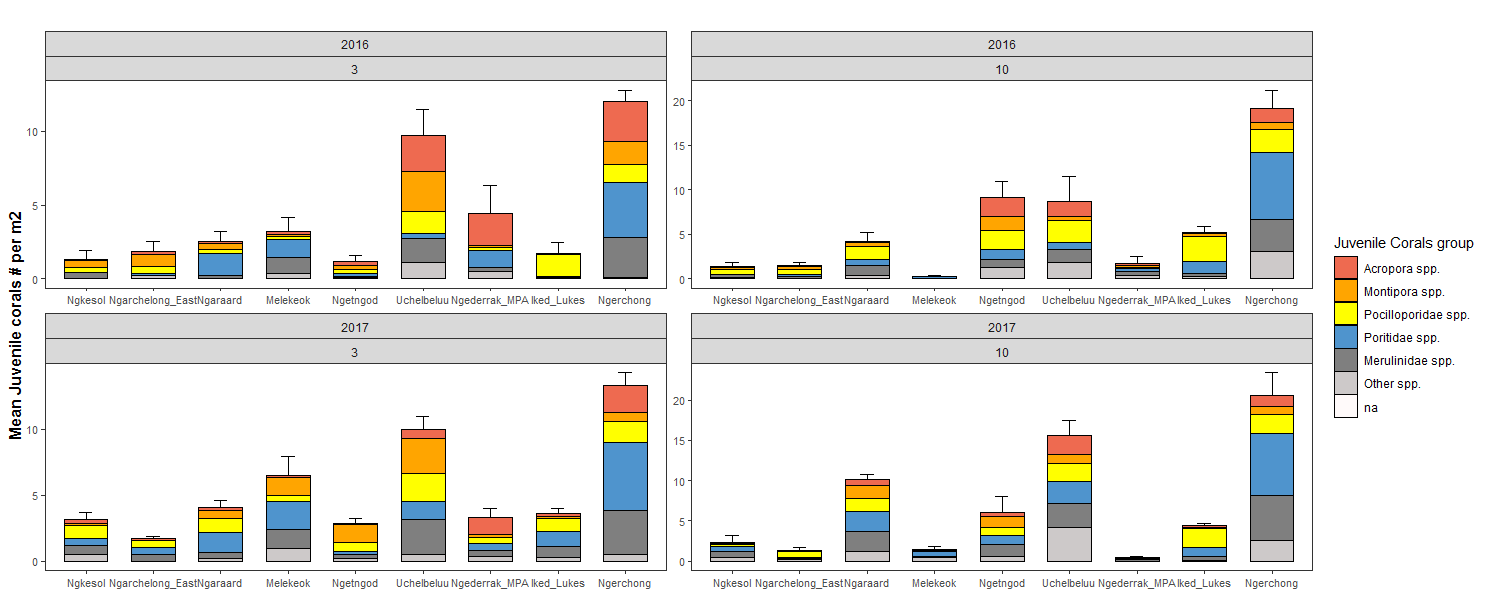


**Figure S5**: Densities of juvenile corals in 2016 and in 2017 at 3 and 10 m depth (n = 5 transects). Error bars show one standard error.

*
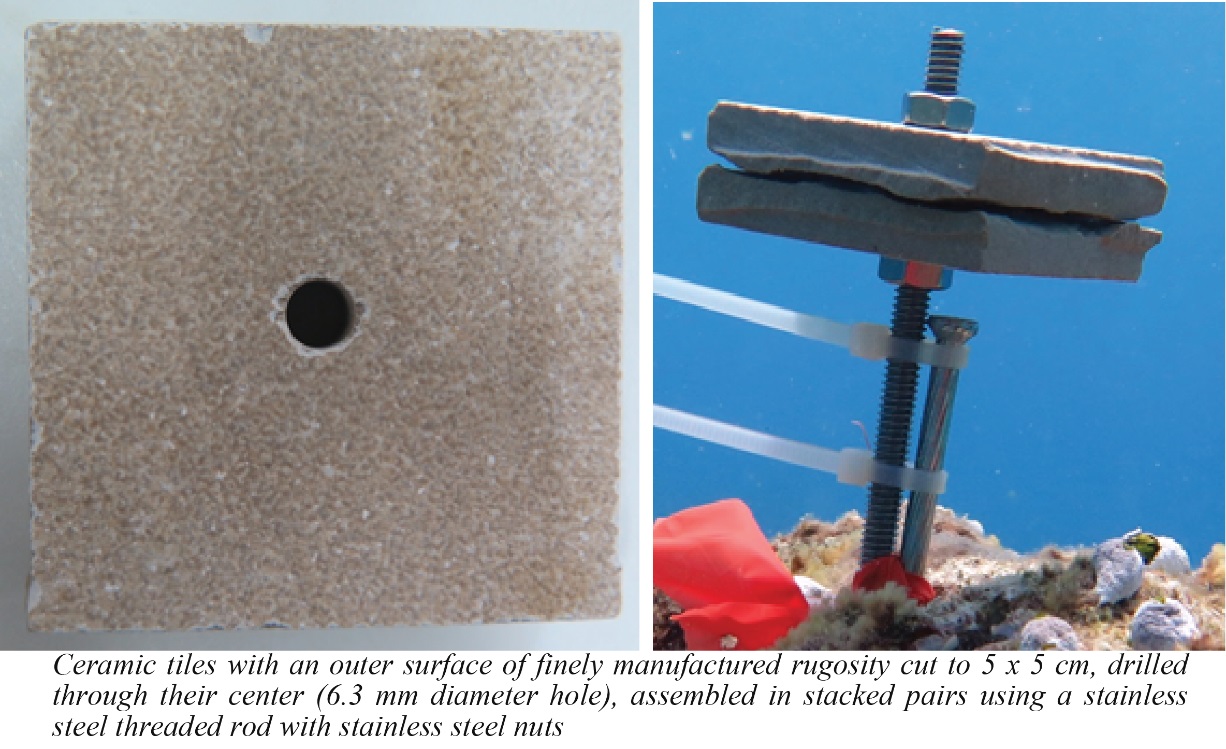
*

**Figure S6:** Photographs of settlement tiles used in the study with rough structured outer surface (left) and a stack of tiles with the rough surface on the outer sides attached on the reefs with concrete nails and cable ties

1. **Supplementary Tables**

**Table S1**: Difference in the total density of settlers among sites and seasons (GLM-NB model)

|  | Df | Deviance | Resid. Df | Resid. Dev | Pr(>Chi) |
| --- | --- | --- | --- | --- | --- |
| NULL |  |  | 790 | 1517.4 |  |
| Sampling Period | 5 | 284.1847 | 785 | 1233.2 | < 0.001 |
| Site | 8 | 414.9612 | 777 | 818.2 | < 0.001 |
| Sampling Period:Site | 40 | 136.603 | 737 | 681.6 | < 0.001 |

**Table S2**: Difference in the total density of juvenile corals between the two years, sites and depths (GLM-NB)

|  | Df | Deviance | Resid. Df | Resid. Dev | Pr(>Chi) |
| --- | --- | --- | --- | --- | --- |
| NULL |  |  | 179 | 1009.17 |  |
| Year | 1 | 12.22 | 178 | 996.94 | < 0.001 |
| Site | 8 | 586.17 | 170 | 410.77 | < 0.001 |
| Depth.m. | 1 | 4.46 | 169 | 406.31 | < 0.05 |
| Year:Site | 8 | 35.70 | 161 | 370.61 | < 0.001 |
| Year:Depth.m. | 1 | 1.15 | 160 | 369.47 | 0.284 |
| Site:Depth.m. | 8 | 152.33 | 152 | 217.14 | < 0.001 |
| Year:Site:Depth.m. | 8 | 24.20 | 144 | 192.94 | < 0.01 |

**Table S3**: Contrasts for differences in juvenile coral densities between year 2016 and 2017 at each site and depth

| Site_Depth | estimate | SE | z.ratio | P value |
| --- | --- | --- | --- | --- |
| Iked_Lukes, 3m | -0.7309 | 0.318 | -2.298 | **0.0216** |
| Melekeok, 3m | -0.7035 | 0.275 | -2.563 | **0.0104** |
| Ngaraard, 3m | -0.4733 | 0.295 | -1.605 | 0.1084 |
| Ngarchelong_East, 3m | 0.0741 | 0.344 | 0.215 | 0.8295 |
| Ngederrak_MPA, 3m | 0.2978 | 0.282 | 1.055 | 0.2916 |
| Ngerchong, 3m | -0.1054 | 0.234 | -0.45 | 0.6526 |
| Ngetngod, 3m | -0.8708 | 0.351 | -2.483 | **0.013** |
| Ngkesol, 3m | -0.8544 | 0.34 | -2.514 | **0.0119** |
| Uchelbeluu, 3m | -0.027 | 0.24 | -0.112 | 0.9104 |
| Iked_Lukes, 10m | 0.1671 | 0.269 | 0.622 | 0.5341 |
| Melekeok, 10m | -1.7047 | 0.583 | -2.925 | **0.0034** |
| Ngaraard, 10m | -0.8807 | 0.258 | -3.411 | **0.0006** |
| Ngarchelong_East, 10m | 0.1466 | 0.377 | 0.389 | 0.6975 |
| Ngederrak_MPA, 10m | 1.4663 | 0.499 | 2.937 | **0.0033** |
| Ngerchong, 10m | -0.0706 | 0.226 | -0.313 | 0.7544 |
| Ngetngod, 10m | 0.4202 | 0.25 | 1.679 | 0.0931 |
| Ngkesol, 10m | -0.5108 | 0.347 | -1.472 | 0.141 |
| Uchelbeluu, 10m | -0.5921 | 0.237 | -2.498 | **0.0125** |

**Table S4**: relationships between juvnile corals abundance at each site and depth in 2017 (different size categories) with settlers density from 2016 and from both years (2016 and 2017)

|  | Df | Sum Sq | Mean Sq | F value | Pr(>F) |
| --- | --- | --- | --- | --- | --- |
| **ACROPORA** | | | | | |
| 1. Acropora 0-2cm |  |  |  |  |  |
| Settlers (2016) | 1 | 0.08468 | 0.084679 | 1.8091 | 0.2 |
| Settlers (2016-2017) | 1 | 0.17772 | 0.177721 | 4.4252 | **0.054.** |
| 1. Acropora 0-3cm |  |  |  |  |  |
| Settlers (2016) | 1 | 0.04263 | 0.042628 | 1.5528 | 0.23 |
| Settlers (2016-2017) | 1 | 0.10048 | 0.100476 | 4.3084 | **0.057.** |
| 1. Acropora 0-4cm |  |  |  |  |  |
| Settlers (2016) | 1 | 0.10551 | 0.105510 | 2.2885 | 0.15 |
| Settlers (2016-2017) | 1 | 0.18975 | 0.189748 | 4.7333 | **0.047*** |
| 1. Acropora 0-5cm |  |  |  |  |  |
| Settlers (2016) | 1 | 0.19621 | 0.196212 | 4.3437 | 0.055 |
| Settlers (2016-2017) | 1 | 0.30191 | 0.301910 | 8.025 | **0.013*** |
| **POCILLOPORIDS** | | | | | |
| 1. Pocilloporids 0-1cm |  |  |  |  |  |
| Settlers (2016) | 1 | 0.00006 | 0.000060 | 0.0011 | 0.9737 |
| Settlers (2016-2017) | 1 | 0.01236 | 0.012358 | 0.2368 | 0.6341 |
| 1. Pocilloporids 0-2cm |  |  |  |  |  |
| Settlers (2016) | 1 | 0.12118 | 0.12118 | 1.0424 | 0.3246 |
| Settlers (2016-2017) | 1 | 0.05265 | 0.052654 | 0.4346 | 0.5204 |
| 1. Pocilloporids 0-3cm |  |  |  |  |  |
| Settlers (2016) | 1 | 0.10491 | 0.10491 | 0.8981 | 0.3594 |
| Settlers (2016-2017) | 1 | 0.03569 | 0.035689 | 0.2931 | 0.5967 |
| **PORITIDS** | | | | | |
| 1. Poritids 0-1cm |  |  |  |  |  |
| Settlers (2016) | 1 | 0.02941 | 0.029414 | 0.3637 | 0.5549 |
| Settlers (2016-2017) | 1 | 0.00191 | 0.001909 | 0.0231 | 0.8811 |
| 1. Poritids 0-2cm |  |  |  |  |  |
| Settlers (2016) | 1 | 0.03429 | 0.034288 | 0.6464 | 0.4332 |
| Settlers (2016-2017) | 1 | 0.00402 | 0.004021 | 0.0732 | 0.7902 |

**References**

1. Jones, G. P. *et al.* Larval retention and connectivity among populations of corals and reef fishes: history, advances and challenges. *Coral Reefs* **28**, 307–325 (2009).

2. Harrison, P. L. Sexual reproduction of scleractinian corals. in *Coral reefs: an ecosystem in transition* 59–85 (Springer, 2011).

3. Connolly, S. R. & Baird, A. H. Estimating dispersal potential for marine larvae: dynamic models applied to scleractinian corals. *Ecology* **91**, 3572–3583 (2010).

4. Friedlander, A. M. & Parrish, J. D. Habitat characteristics affecting fish assemblages on a Hawaiian coral reef. *J. Exp. Mar. Biol. Ecol.* **224**, 1–30 (1998).

5. Gratwicke, B. & Speight, M. R. The relationship between fish species richness, abundance and habitat complexity in a range of shallow tropical marine habitats. *J. Fish Biol.* **66**, 650–667 (2005).

6. Graham, N. A. J., Wilson, S. K., Pratchett, M. S., Polunin, N. V. C. & Spalding, M. D. Coral mortality versus structural collapse as drivers of corallivorous butterflyfish decline. *Biodivers. Conserv.* **18**, 3325–3336 (2009).

7. Doropoulos, C., Roff, G., Visser, M.-S. & Mumby, P. J. Sensitivity of coral recruitment to subtle shifts in early community succession. *Ecology* **92**, 304–314 (2017).

8. Mundy, C. N. & Babcock, R. C. Role of light intensity and spectral quality in coral settlement: implications for depth-dependent settlement? *J. Exp. Mar. Biol. Ecol.* **223**, 235–255 (1998).

9. Raimondi, P. T. & Morse, A. N. The consequences of complex larval behavior in a coral. *Ecology* **81**, 3193–3211 (2000).

10. Baird, A. H. & Morse, A. N. Induction of metamorphosis in larvae of the brooding corals Acropora palifera and Stylophora pistillata. *Mar. Freshw. Res.* **55**, 469–472 (2004).

11. Morse, D. E. & Morse, A. N. Enzymatic characterization of the morphogen recognized by Agaricia humilis (scleractinian coral) larvae. *Biol. Bull.* **181**, 104–122 (1991).

12. Babcock, R. & Mundy, C. Coral recruitment: consequences of settlement choice for early growth and survivorship in two scleractinians. *J. Exp. Mar. Biol. Ecol.* **206**, 179–201 (1996).

13. Antonius, A. Pneophyllum conicum, a coralline red alga causing coral reef-death in Mauritius. *Coral Reefs* **19**, 418–418 (2001).

14. Birrell, C. L., McCook, L. J., Willis, B. L. & Diaz-Pulido, G. Effects of benthic algae on the replenishment of corals and the implications for the resilience of coral reefs. *Ocean. Mar Biol Annu Rev* **46**, 25–63 (2008).

15. Diaz-Pulido, G., Harii, S., McCook, L. J. & Hoegh-Guldberg, O. The impact of benthic algae on the settlement of a reef-building coral. *Coral Reefs* **29**, 203–208 (2010).

16. Arnold, S., Steneck, R. & Mumby, P. Running the gauntlet: inhibitory effects of algal turfs on the processes of coral recruitment. *Mar. Ecol. Prog. Ser.* **414**, 91–105 (2010).

17. Leigh, E. G., Paine, R. T., Quinn, J. F. & Suchanek, T. H. Wave energy and intertidal productivity. *Proc. Natl. Acad. Sci.* **84**, 1314–1318 (1987).

18. Roff, G. *et al.* Exposure-driven macroalgal phase shift following catastrophic disturbance on coral reefs. *Coral Reefs* **34**, 715–725 (2015).

19. Roff, G. *et al.* Phase shift facilitation following cyclone disturbance on coral reefs. *Oecologia* **178**, 1193–1203 (2015).

20. Doropoulos, C. *et al.* Reef-scale failure of coral settlement following typhoon disturbance and macroalgal bloom in Palau, Western Pacific. *Coral Reefs* **33**, 613–623 (2014).

21. Bejarano, S. *et al.* The shape of success in a turbulent world: Wave exposure filtering of coral reef herbivory. *Funct. Ecol.* **31**, 1312–1324 (2017).

22. Diaz-Pulido, G. & McCook, L. J. The fate of bleached corals: patterns and dynamics of algal recruitment. *Mar. Ecol. Prog. Ser.* **232**, 115–128 (2002).

23. Hughes, T. P. *et al.* Phase shifts, herbivory, and the resilience of coral reefs to climate change. *Curr. Biol.* **17**, 360–365 (2007).

24. Smith, J. E., Hunter, C. L. & Smith, C. M. The effects of top–down versus bottom–up control on benthic coral reef community structure. *Oecologia* **163**, 497–507 (2010).

25. Steneck, R., Arnold, S. & Mumby, P. Experiment mimics fishing on parrotfish: insights on coral reef recovery and alternative attractors. *Mar. Ecol. Prog. Ser.* **506**, 115–127 (2014).

26. Diaz-Pulido, G. & McCook, L. J. Relative roles of herbivory and nutrients in the recruitment of coral-reef seaweeds. *Ecology* **84**, 2026–2033 (2003).

27. Doropoulos, C., Ward, S., Marshell, A., Diaz-Pulido, G. & Mumby, P. J. Interactions among chronic and acute impacts on coral recruits: the importance of size-escape thresholds. *Ecology* **93**, 2131–2138 (2012).

28. Mumby, P. J. Herbivory versus corallivory: are parrotfish good or bad for Caribbean coral reefs? *Coral Reefs* **28**, 683–690 (2009).

29. Venera-Ponton, D. E., Diaz-Pulido, G., McCook, L. J. & Rangel-Campo, A. Macroalgae reduce growth of juvenile corals but protect them from parrotfish damage. *Mar. Ecol. Prog. Ser.* **421**, 109–115 (2011).
